# Correlations between lignin content and structural robustness in plants revealed by X-ray ptychography

**DOI:** 10.1038/s41598-020-63093-6

**Published:** 2020-04-07

**Authors:** Carla C. Polo, Luciano Pereira, Paulo Mazzafera, Denisele N. A. Flores-Borges, Juliana L. S. Mayer, Manuel Guizar-Sicairos, Mirko Holler, Mariane Barsi-Andreeta, Harry Westfahl, Florian Meneau

**Affiliations:** 1 Brazilian Synchrotron Light Laboratory (LNLS), Brazilian Center for Research in Energy and Materials (CNPEM), 13083-970 Campinas, SP Brazil; 2 Laboratory of Plant Physiology “Coaracy M. Franco”, Center R&D in Ecophysiology and Biophysics, Agronomic Institute (IAC), Campinas, SP Brazil; 3 Department of Plant Biology, Institute of Biology, P.O. Box 6109, University of Campinas (UNICAMP), 13083-970 Campinas, SP Brazil; 4 Departament of Crop Science, College of Agriculture “Luiz de Queiroz”, University of São Paulo (ESALQ-USP), CP 09, 13418-900 Piracicaba, SP Brazil; 5 Paul Scherrer Institute, Villigen, PSI Switzerland; 6 São Carlos Institute of Physics, University of São Paulo, PO Box 369, 13560-970 São Carlos, SP Brazil

**Keywords:** Cell wall, Crop waste

## Abstract

Lignin is a heterogeneous aromatic polymer responsible for cell wall stiffness and protection from pathogen attack. However, lignin represents a bottleneck to biomass degradation due to its recalcitrance related to the natural cell wall resistance to release sugars for fermentation or further processing. A biological approach involving genetics and molecular biology was used to disrupt lignin pathway synthesis and decrease lignin deposition. Here, we imaged three-dimensional fragments of the petioles of wild type and C4H lignin mutant *Arabidopsis thaliana* plants by synchrotron cryo-ptychography. The three-dimensional images revealed the heterogeneity of vessels, parenchyma, and fibre cell wall morphologies, highlighting the relation between disturbed lignin deposition and vessel implosion (cell collapsing and obstruction of water flow). We introduce a new parameter to accurately define cell implosion conditions in plants, and we demonstrate how cryo-ptychographic X-ray computed tomography (cryo-PXCT) provides new insights for plant imaging in three dimensions to understand physiological processes.

## Introduction

Lignin is a hydrophobic and heterogeneous biopolymer fundamental for the development of an efficient water transport system in plants, conferring structural robustness and impermeability to conduits, essential for plant stiffness^1,2^. Lignin is found in the plant cell wall, mainly in vessels and fibres, forming chemical bonds with hemicellulose, which adheres to cellulose microfibrils. This polymer plays a fundamental physiological role during pathogen infection by inducing cell wall coarsening, impeding the action of fungal and bacterial cellulolytic enzymes and consequently inhibiting the pathogen invasion of surrounding tissues^3^. On the other hand, for lignocellulosic biofuel production, lignin is among the molecules that limit the value of biomass crops, impacting the cellulose breakdown to glucose, which is used for further fermentation steps^4,5^.

Different pretreatment approaches have been developed to change the physical and chemical nanostructure of lignocellulosic biomass to alter its three-dimensional structure, interactions and composition to improve hydrolysis rates^6–10^. As an alternative, genetic manipulations of the lignin biosynthetic pathway can alter the composition and reduce the content of lignin, thereby decreasing biomass recalcitrance related to the natural cell resistance to release sugars for fermentation or further processing. This effect has been largely elucidated for both *Arabidopsis thaliana*, a model organism for plants, and other species^1,11–15^. However, the compromised growth of manipulated plants has made the commercial use of these crops a problematic issue, since the lack of cellular rigidity makes the vessels more susceptible to embolism formation^11,16,17^ or collapse (i.e., conduit implosion)^18^, preventing the water transport along the plant.

Therefore, it is crucial to determine the three-dimensional distribution of lignin in the cell walls and in different tissues to understand how the cell wall monomeric composition varies^19^. Structural analysis via optical microscopy and X-ray scanning microdiffraction provided insights in terms of two-dimensional imaging of collapsed vessels to characterise the relationship between the decrease in lignin depositions and disturbance in cellulose microfibrils orientation^20,21^. However, there is a lack of information regarding the whole cellular volumetric scenario: indeed, whether the mutation effects are homogeneous along the entire conducting vessel secondary wall and how these mutations affect other cellular types remain open questions.

Several imaging techniques have provided three-dimensional information on plant cell anatomy. Focused ion beam-scanning electron microscopy (FIB-SEM) allowed us to study the architecture of 20 µm^3^ fragments of *Arabidopsis*, creating three-dimensional renderings of organelle identification^22^. Confocal microscopy^23^, X-ray computed tomography^24^ and transmission electron microscopy (TEM)^25^ opened the path to obtain high resolution images of biological systems. However, these techniques present shortcomings with respect to compulsory sample labelling, the small field of view and the limited sample volume and/or the excessively long measurement times. Nevertheless, the availability of coherent hard X-rays from synchrotron light sources opens new opportunities for plant bio-imaging.

Ptychographic X-ray computed tomography (PXCT) is a lens-less imaging technique that can reveal the three-dimensional hierarchical structure of biomaterials over large fields of view, with quantitative electron density contrast^26,27^. A real space image of the electron density distribution is retrieved through iterative algorithms^28^, applied to precise measurements of coherent X-rays scattered by the specimen, ultimately reaching spatial resolutions on the order of 10 nm in the case of strongly scattering materials^29,30^. PXCT was applied to unveil the nanometric features of stain-free biological samples, on single cells and within tissues, resolving organelles and macromolecules^31,32^. Here, we obtained a three-dimensional image of plant tissues corresponding to volumes of 25 ×50 ×50 μm^3^ of *Arabidopsis thaliana*, which is considered a model organism for plants.

We used PXCT under cryogenic conditions to prevent radiation damage and investigate the effects of differential lignin deposition between the *Arabidopsis thaliana* wild type genotype and the cinnamate 4-hydroxylase enzyme (C4H) deletion phenotype. The downregulation of the *C4H* gene leads to a drastic lignin reduction, and the plants display severe dwarfism and sterility^21,33^. A direct comparison between the three-dimensional images of the two genotypes unveils morphological dissimilarities that correlate directly with the lignin content and its structural role in plants. A quantitative analysis suggests a strong link between the probability distributions of the cell wall/lumen volume together with thickness along the entire cellular extension and structural robustness. We demonstrate that the narrower dispersion and the smaller wall thickness in the mutant cells correlate with an increased susceptibility to cell implosion with respect to the wild type cells.

## Results

### Three-dimensional nanoscale imaging of *Arabidopsis thaliana* plant tissue

**Figure 1:**
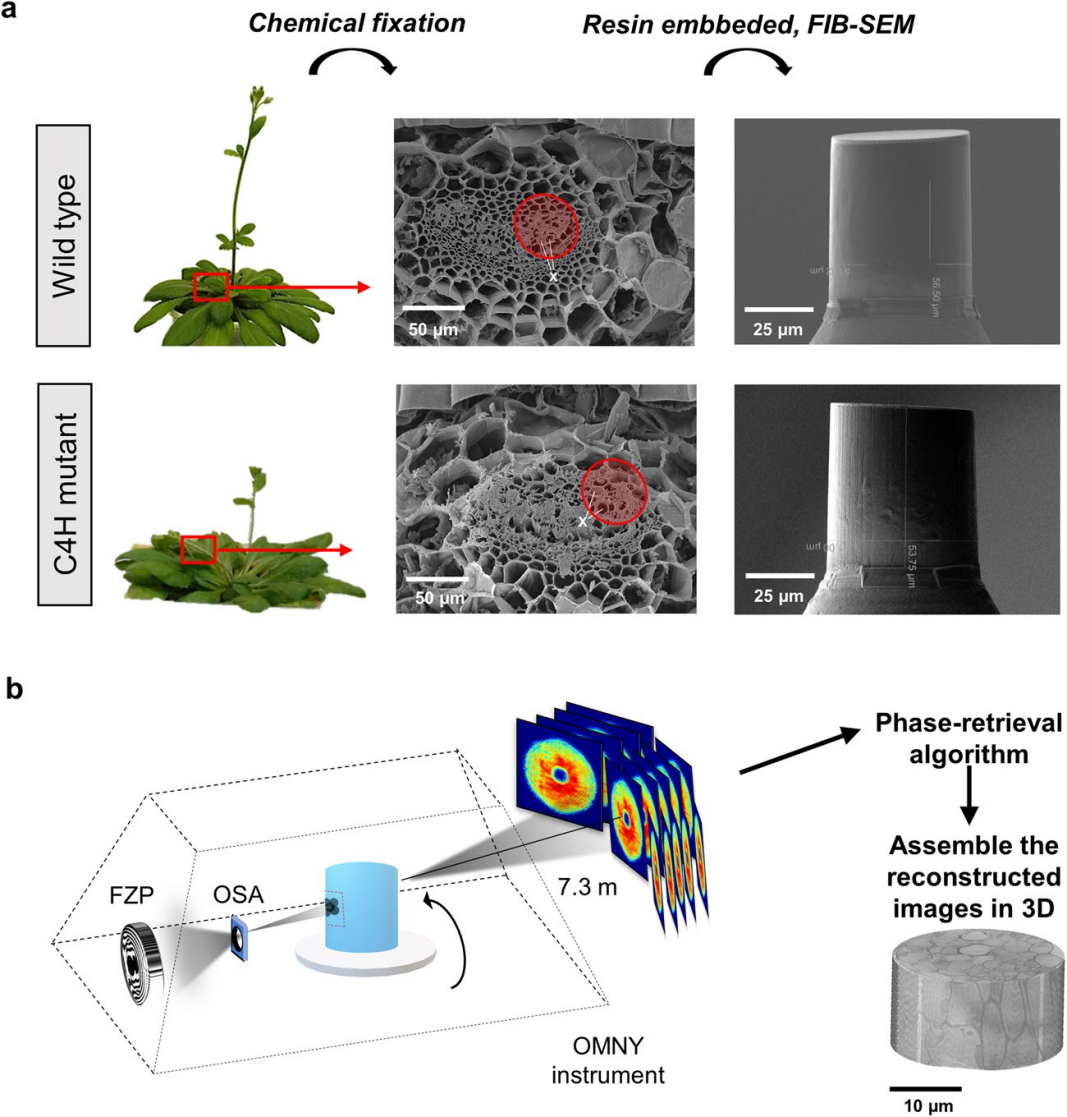
PXCT experiment workflow. (**a**) The petioles of wild type and C4H mutant plants were selected (red square), and the overall petiole structure was measured by scanning electron microscopy to identify regions containing vessels (X). The tissue fragments were submitted to chemical fixation, and resin infusion and the 50 µm diameter x 50 µm height regions of interest (red circle) were cut out using FIB-SEM. (**b**) OMNY instrument used in the cSAXS beamline (PSI) for the X-ray experiment. The sample was inserted into the OMNY cryogenic device^34^, which uses a Fresnel zone plate (FZP) to focus and define the coherent beam size. A combination of a central stop (CS) and an order sorting aperture (OSA) are used as filters, such that only the first diffraction order makes it to the sample. This removes additional diffraction orders as well as the undiffracted component of the beam. In the ptychographic measurements, the sample was scanned with overlapping coherent X-ray illumination for each tomographic projection between 0 and 180°. Each two-dimensional diffraction pattern is recorded in the far-field and the phase is retrieved by iterative algorithms that reconstruct a real-space two-dimensional projection. The set of two-dimensional projections are then used to reconstruct the tomographic image.

The wild type and C4H mutant of *Arabidopsis thaliana* cv. Columbia plants were imaged with cryo-ptychographic X-ray computed tomography (cryo-PXCT). The plant specimens (see Methods) were cut using focused ion beam milling (FIB) and shaped as pillars of 50 μm in height and 50 μm in diameter. Figure 1a presents the sample preparation workflow with scanning electron microscopy images of the wild type and C4H mutant *Arabidopsis thaliana* plants and the corresponding FIB-shaped pillars. The cryo-PXCT measurements were conducted at the cSAXS beamline of the Swiss Light Source at Paul Scherrer Institute (PSI), and a schematic representation of the setup is presented in Fig. 1b. Experimentally, during X-ray ptychography measurements, the scattering patterns are measured in the far field regime at several sample positions in the x–y plane with mutually overlapping X-ray illumination. Iterative phase retrieval algorithms are applied to reconstruct the complex-valued transmissivity of the specimen^26^. By combining the data acquisition at different angles, we obtained tomographic reconstructions that provide three-dimensional reconstructions with nanometric precision^27,30–32^.

**Figure 2:**
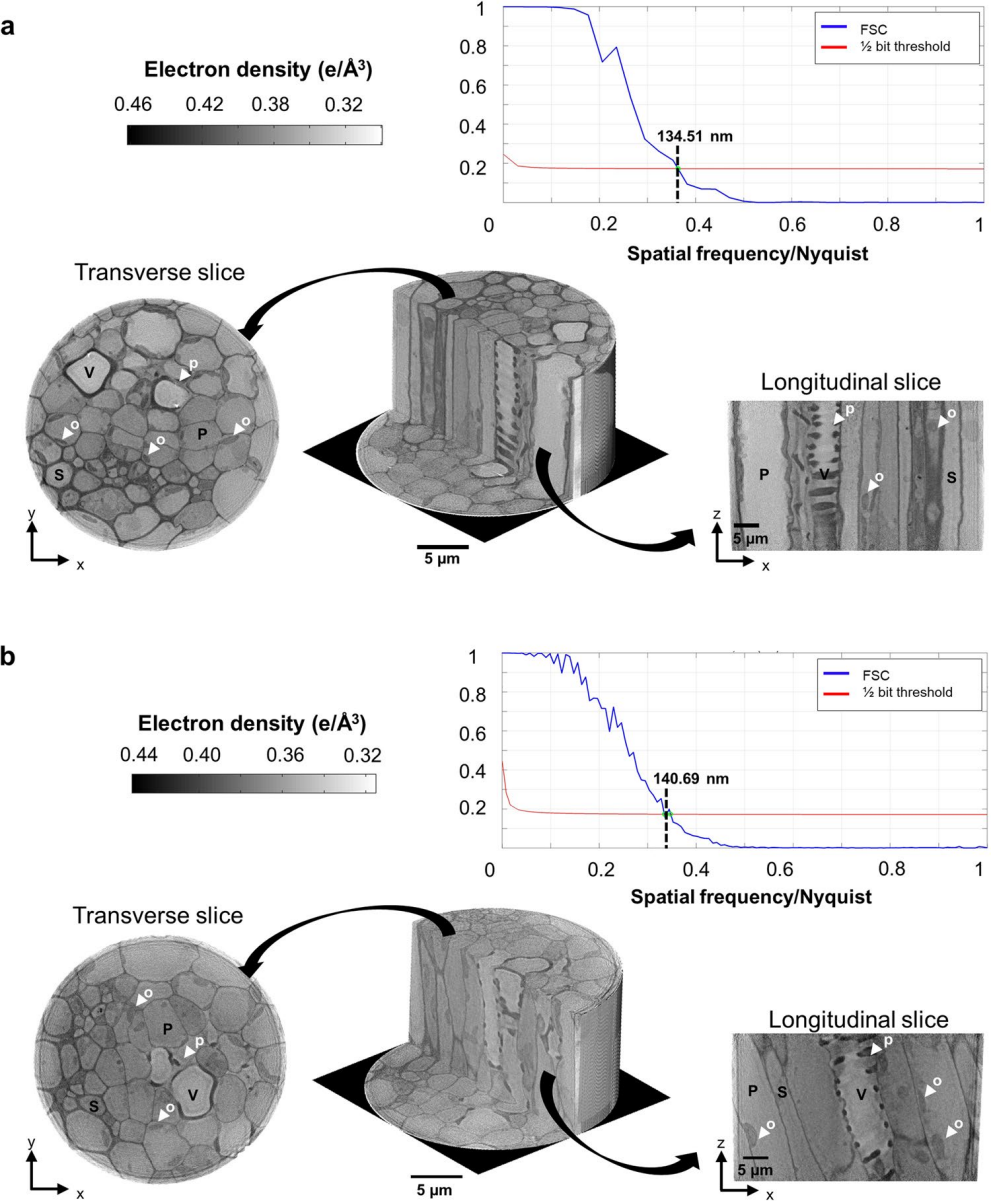
Tomographic phase reconstructions of *A. thaliana* petiole fragments. 3D reconstructions of the wild type tissue **(a),** and the C4H mutant tissue **(b)**. The tomograms were converted to electron density (e/Å^−3^), where the lower values are white and higher values are dark shades of grey. The different views are depicted by the tomogram transversal (x,y) and longitudinal (x, z) slices, highlighting the different cellular types: the vessel (V), sclerenchyma (S) and parenchyma (P) cells. Intracellular structures, such as organelles (o) in parenchymatic cells and pits (p) in vessel cells, are also indicated by the white arrows. The Fourier shell correlation (FSC) is used to estimate the spatial resolution for both reconstructed tomograms based on the half bit threshold criteria, resulting in 134 nm and 140 nm 3D resolution for the wild type and C4H mutant plant fragments, respectively.

The voxel size of the reconstructed cryo-PXCT three-dimensional images in real space was as small as 49 nm, in contrast to the volume of the plant specimen of 50 μm^3^ thick, enabling the determination of the 3D hierarchical structure of the plant specimen, tracking cell wall thickness variations and the cell/lumen diameter distribution between wild type and C4H mutant *Arabidopsis thaliana* plants. Figure 2 shows the three-dimensional reconstruction of the wild type and C4H mutant plants, revealing their multiscale structures and highlighting the resulting cross-sectional images, which correspond to transverse (x,y) and longitudinal (x,z) slices. The colour of the tomograms corresponds to the electron density maps of the different cellular compartments. The reconstructed images reach spatial resolutions of 134 nm and 140 nm, as determined by Fourier shell correlation^35^ for the wild type and C4H mutant plants, respectively (Fig. 2).

From these cryo-PXCT images (Fig. 2), we can identify the main cellular types, such as vessels (conduits), sclerenchyma cells (support fibres) and parenchyma cells (nutrient storage), by their anatomical morphologies. The vessels show a large lumen and helicoidal or scalariform region in the cell wall. The sclerenchyma, usually dead cells with a structural role, display the typical thick cell walls with a cylindrical small lumen. Because conduit vessels are dead cells, these cells present no cytoplasm in plants *in vivo*; therefore, the lumen is filled with resin in these images. Finally, the parenchymatic cells possess a characteristic cylindrical shape with a thin cell wall. The cytoplasm contains large vacuoles filled with water and dissolved substances, such as lipids, depicting the different cellular compartments.

### Morphology and thickness distribution provided by segmentation analysis

The digital segmentation of the cellular compartments using machine learning was based in manual pixel annotations in order to train a classifier and segment the remaining data automatically^36^ followed by grey level threshold (for more details see Methods).

**Figure 3:**
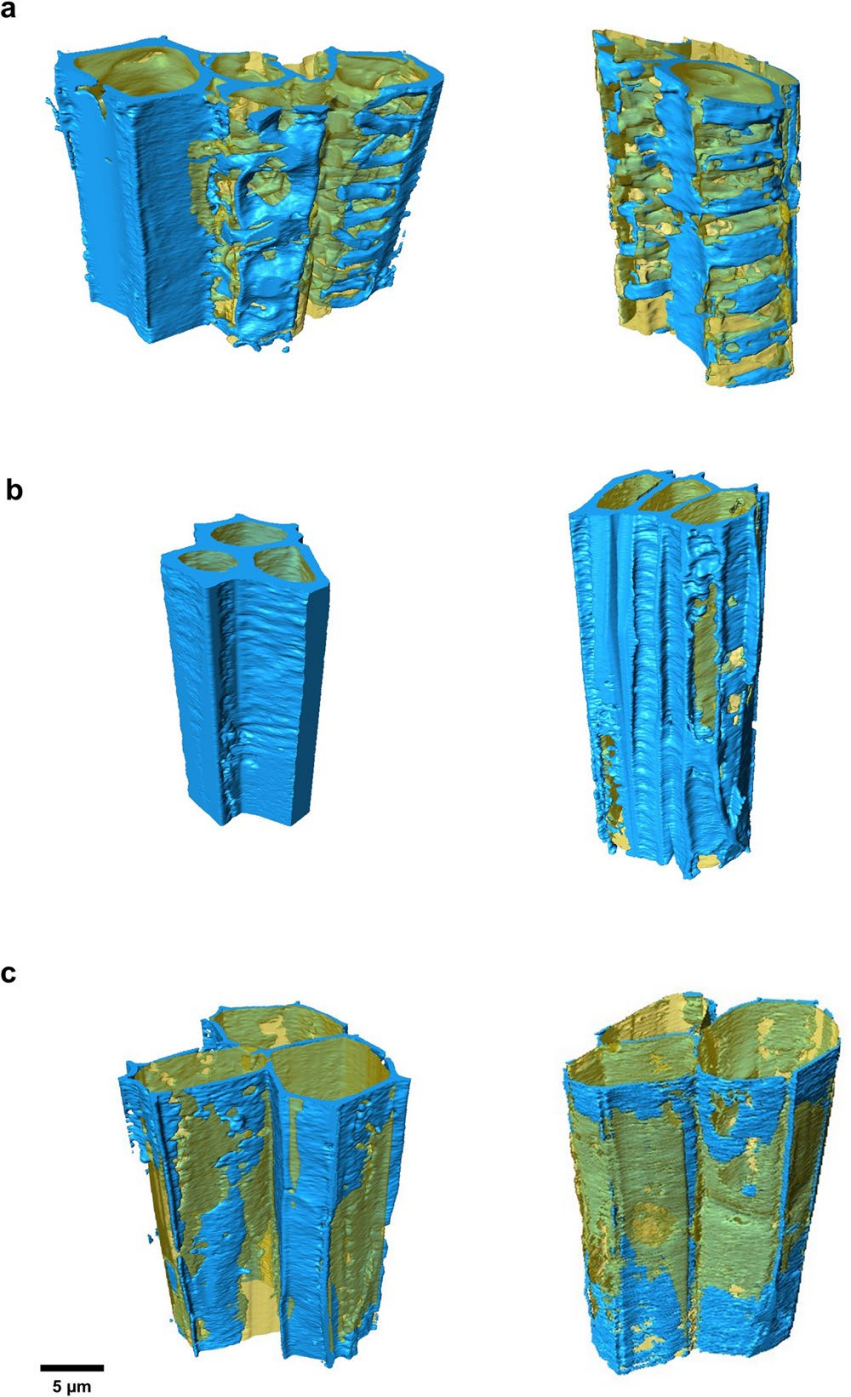
Segmentation and 3D rendering of different cellular types. The vessel (**a**), sclerenchyma (**b**), and parenchyma (**c**) cells (*N* = 3) were extracted from the measured block and segmented using a machine learning approach. The cell wall (blue) and lumen (yellow) were separated, showing the heterogeneous character of these cellular compartments. The morphological dissimilarities between wild type cells (left) and C4H mutant cells (right) are also evident in these images.

The final segmented image enabled us to separately quantify the thickness and volume of the cell wall and lumen (empty cell interior for vessel and sclerenchyma cells or filled with cytoplasm in the parenchyma cells) (Fig. 3). With quantitative information from the different cellular types, we could identify the anatomical changes presented by the mutant plant cells compared to the wild type plant cells.

Most of the lignin deposition during plant development occurs in sclerenchyma, which are the supporting fibres, and in vessel tissue, that also has a whole of structural support, besides the water and nutrients transport^3^.

**Figure 4:**
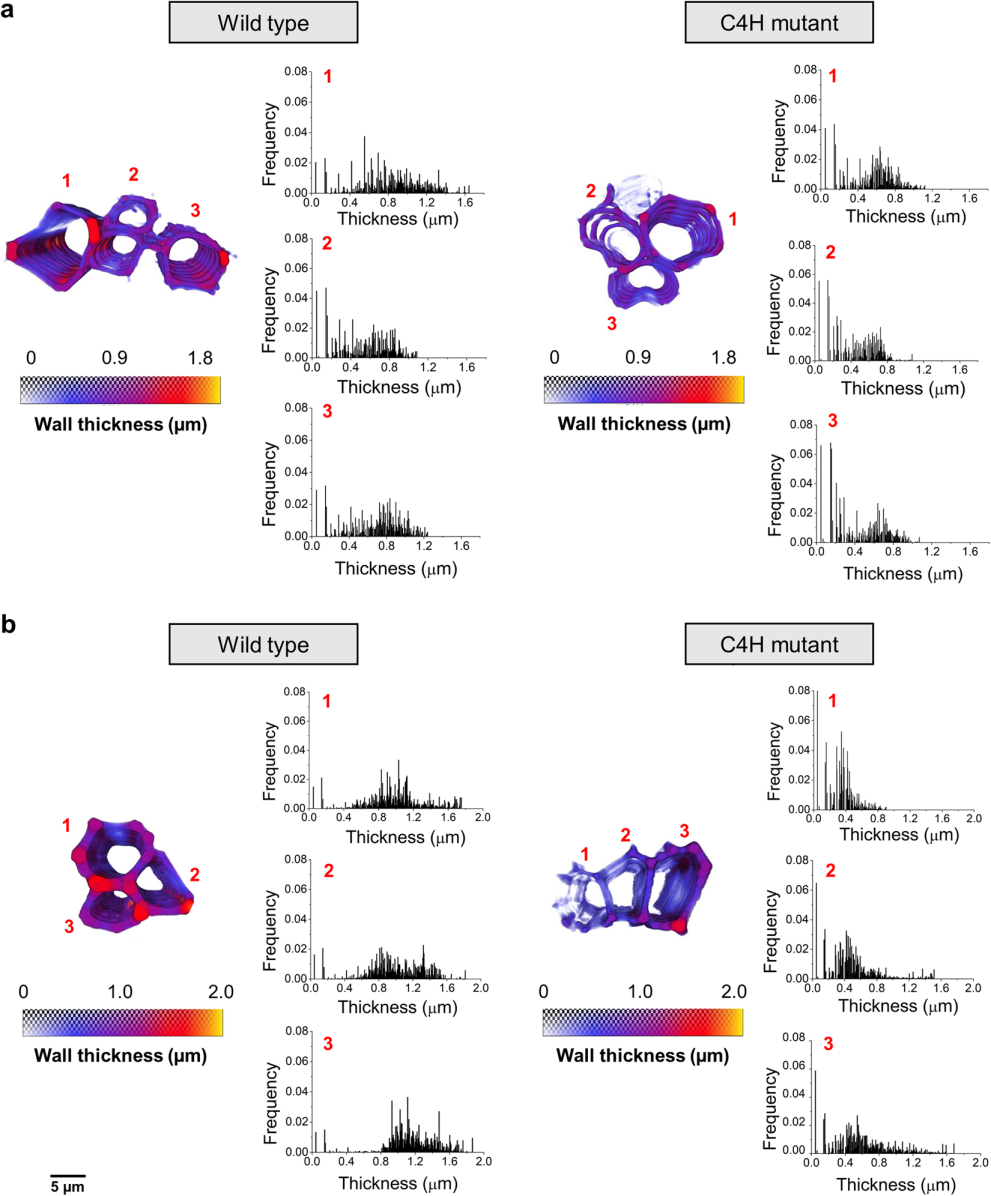
Cell wall thickness distributions of vessel and sclerenchyma cells. Analysis of the segmented vessel (**a**), and sclerenchyma (**b**), cells allowed us to observe the wall distribution and evaluate the differences in thickness between wild type (left) and C4H mutant (right) tissue fragments. The colour maps of each cell block represent the three-dimensional maps of the thickness distribution along the cell wall volume. The colour map goes from smaller thickness (violet) to larger thickness (yellow), with the values, in micrometres, highlighting the differences between wild type and mutant plants. The wall thickness analysis of each cell numbered from 1 to 3 corresponds to the histograms of thickness (µm) *versus* frequency.

We used local thickness 3D measurement^37^, implemented in Avizo (Thermo Scientific™), to obtain the distribution maps providing vessel and sclerenchyma wall thickness (Fig. 4) and respective lumen diameter variations along the entire volume (see Supplementary Figs. S1–S3). The maps are represented by the violet gradient, from dark shades (thinner) to faded shades (thicker).

The corresponding wall thickness distribution histograms are shown in Fig. 4. We observed a 25% decrease in the average vessel cell wall thickness, from 0.8 to 0.6 µm between the wild type and C4H mutant. This decrease, which is larger than our experimental resolution, is also accompanied by a narrowing distribution of the cell wall thickness, while the diameter and distribution of the vessel lumen (see Supplementary Fig. S1) remain essentially unchanged. The most striking differences concerns the sclerenchyma cells (Fig. 4b), which exhibit a two-fold reduction in the cell wall thickness between the wild type and C4H mutant, decreasing from 1 µm to 0.45 µm. This dramatic cell wall reduction is also followed by a narrowing of the wall thickness distribution, as shown in Fig. 4b, whereas the vessel lumen diameter was only slightly reduced (see Supplementary Fig. S2).

The parenchyma cell walls do not present major differences (see Supplementary Fig. S4). Indeed, parenchyma cells possess less secondary growth in their cell walls, and thus, mutation effects are not expected in this cell type.

The three-dimensional cell wall thicknesses and lumen diameter distributions are best visualised in the supplementary videos (see Supplementary Videos S1–S6), displaying the anatomical changes induced by the gene mutation throughout the entire volume of the wild type and mutant plants. The PXCT image segmentations establish a clear correlation with the wall thickness thinning of sclerenchyma cells, which is directly affected by the decrease in guaiacyl-derived lignins (G) induced by the absence of the C4H enzyme. Previous findings determined by derivatisation, followed by the reductive cleavage (DFRC) method, have shown that the decrease in total lignin in the C4H mutant appears to be caused exclusively by a reduction in G subunit content, leading to a substantial increase in the mole percentage of syringyl (S) subunits in the lignin of the mutant^21,38,39^. This finding is in agreement with the cell wall electron density differences observed between the wild type and the mutant plant and showing that both vessel and sclerenchyma cells are affected by the decrease in G-lignins induced by the absence of C4H enzyme.

The distinct cell functions of fibres and vessels in angiosperms and their different S/G ratios are good indicators of functional control of lignin monomer deposition. The increase in S units suggests a more elastic polymer as a result of a lower abundance of linkages among lignin monomers^40^, which may influence the hydraulic as well as the mechanical properties of the cell wall, impacting the cell wall rigidity, and may lead to cell collapse^18^.

### Computing the heterogeneity in implosion resistance parameter

The water transport in plants occurs by negative pressure: the vessel cell wall to lumen relationship is the parameter that describes the resistance of the conduit to implosion^41,42^. The theoretical vessel implosion resistance parameter (*t*/*b*)^2^ is calculated based on the double intervessel wall thickness, *t*, divided by the maximum diameter of the vessel, *b*^41^. The greater the reinforcement against collapse from bending, the higher the *t*/*b* ratio is and the more mechanically resistant the cylinder is to transverse buckling^43^. To date, *b* is estimated as the side of a square with an area equal to the average conduit lumen, and *t* is measured directly based on digital two-dimensional images^41^. The latter method lacks precision, statistics and reproducibility, whereas our three-dimensional image analysis can precisely measure the cell wall thickness, lumen diameter and their distributions to establish the *t*/*b* ratio as a function of the cellular height. Based on the three-dimensional image analysis, we demonstrated that the minimum and maximum *t* and *b* found in a single vessel should be taken into account to determine the implosion resistance parameter instead of an averaged value.

**Figure 5:**
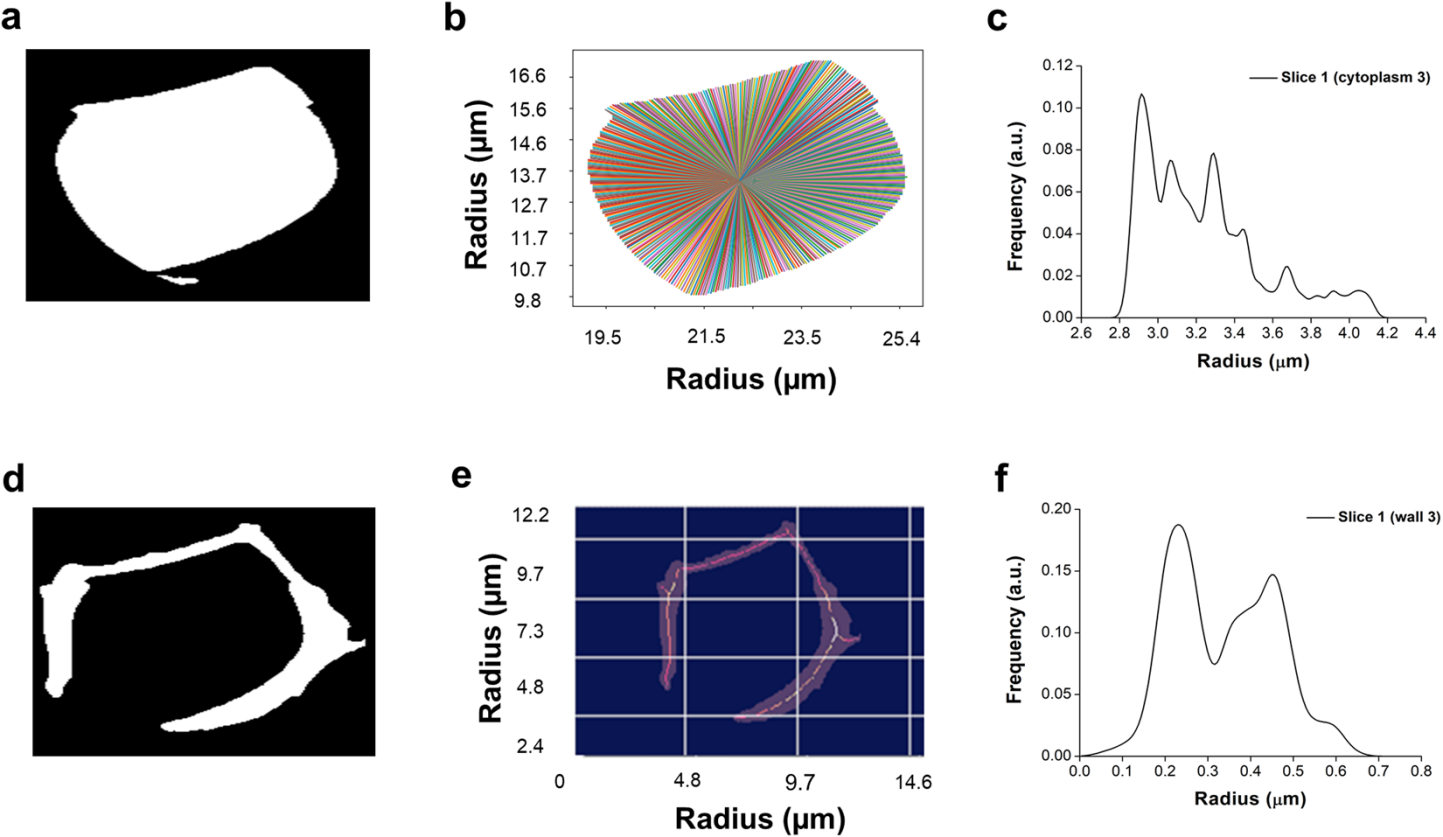
Vessel lumen diameter and cell wall thickness measurements at a given height using the Euclidean distance transform algorithm (EDT). Lumen (**a**), and cell wall (**d**), tomogram slices were preprocessed and binarised. The lumen diameter was measured by acquiring the distance of the centre pixel to all the border pixels (**b**), and different radius measurements are shown in (**c**), together with the frequencies. The cell wall thickness (**e**) is obtained by measuring the distance of the medial axis pixels to the border pixels with all the radius measurements represented in (**f**) by their frequencies.

The Euclidean distance transform algorithm (EDT)^44^ enabled statistically accurate measurements from the vessel lumen diameter and cell wall thickness along the cell heights based on the 2D virtual tomogram slices. To extract the lumen diameters (Fig. 5a) the algorithm sets the minimum distance of each pixel from the border pixels of the object of interest. The centre of the object is taken as the position of the maximum of the distance transform data. The diameter is measured by acquiring the distance of the centre pixel to all the border pixels (Fig. 5b and c). The cell wall images were also binarised, and the EDT method was applied (Fig. 5d). A thinning process is used to extract the median axis of the wall images. The thickness is thus obtained by measuring the distance of the median axis pixels to the border pixels (Fig. 5e, f).

**Figure 6:**
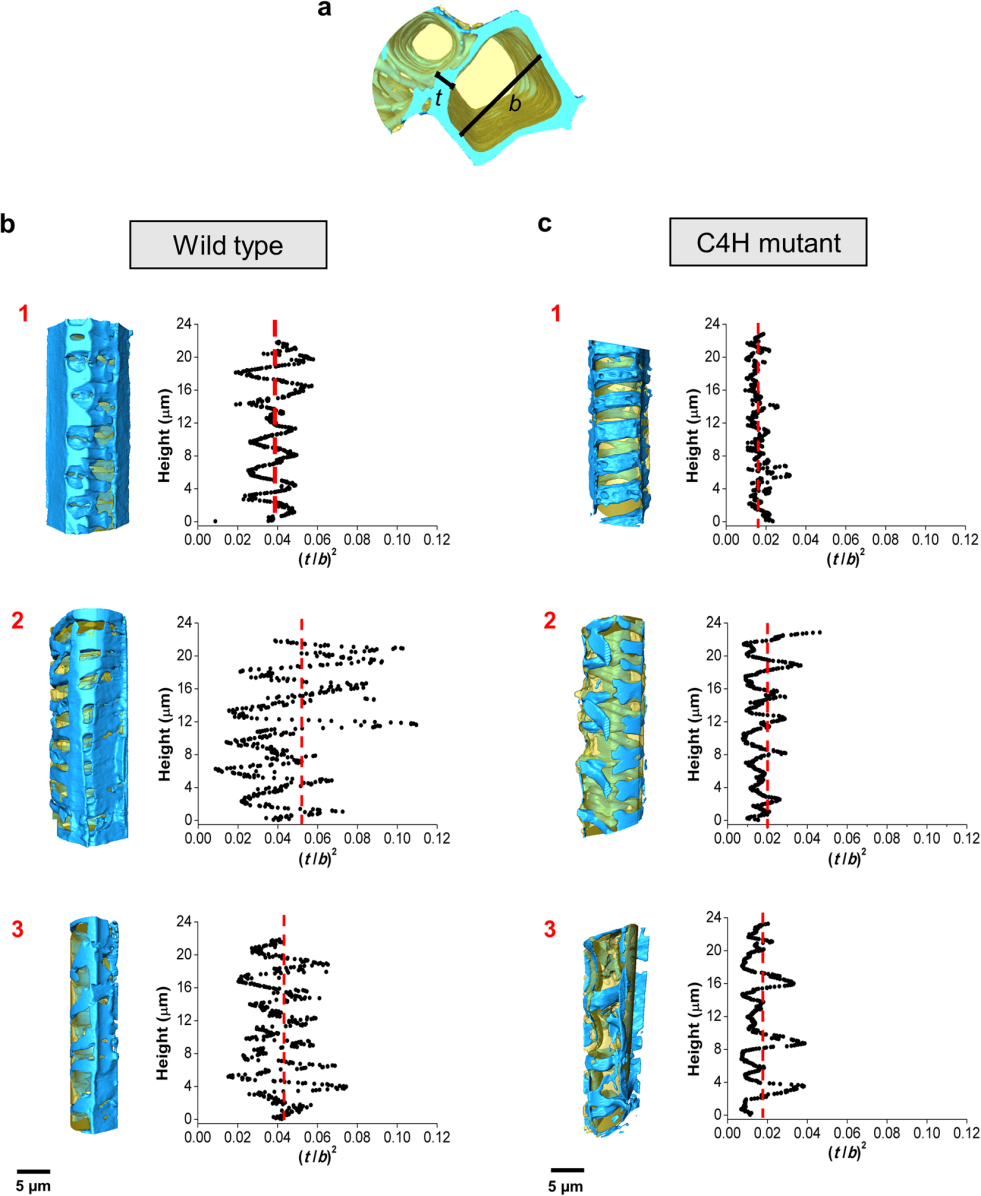
Vessel implosion resistance analysis. (**a**) rendering indicating the structures selected for the analysis, the double wall thickness, *t*, and the lumen diameter, *b*, measured by the EDT algorithm to calculate the implosion resistance along the cellular height. The vessel cells of wild type (**b**), and C4H mutant plants (**c**) were individualised (rendering on the left) and the implosion resistance parameter, (*t*/*b*)^2^, was calculated and plotted according to the cellular heights (right), for each cell (1 to 3). The red dotted line represents the average (*t*/*b*)^2^ showing the displacement to lower values from wild type to the mutant cells.

We measured *t* and *b* in intervals of 50 nm and calculated (*t*/*b*)^2^ along the cellular height. Figure 6 presents (*t*/*b*)^2^ as a function of cellular height for the wild type and mutant plants. The implosion resistance parameter of the wild type vessel cells presents wide variations, with an average value of 0.04 and ranging from 0.002 to 0.100, which is in good agreement with previous work reported for angiosperms^41^. For the mutant, the implosion resistance parameter range shrinks considerably, between 0.006 to 0.040, with an average value of 0.019, which is less than half of the wild type resistance parameter. The lower values of the C4H mutant indicate that most of the cell wall regions may implode under drought conditions when the water pressure is between −1.8 MPa [(*t*/*b*)^2^ = 0.006) and −3 MPa ((*t*/*b*)^2^ = 0.01], which is not uncommon to observe in water-stressed plants^45,46^.

## Discussion

The implosion resistance analysis shows the distribution of (*t*/*b*)^2^ in individual vessel cells. Although the wild type vessel cells present regions with lower (*t*/*b*)^2^ values compared to those in mutant cells, their frequencies are very different. The low amplitude of (*t*/*b*)^2^ distribution in the mutant cells may be the key factor inducing cell implosion. Naturally, cell wall variation exists due to orifices (i.e., pits). Nevertheless, the vessel does not collapse at normal pressures, i.e. when there is no water deficit in the soil and the transport occurs under low pressure. The vessel wall and fibre cell wall thickness are also involved in implosion and embolism resistances since reinforced vessel cell walls may avoid nucleation from which micro-cracks could occur^41,47,48^. Indeed, the surrounding tissues, such as fibres, also act to strengthen vessel walls, increasing resistance to implosion without necessarily changing in either the vessel wall thickness or the lumen diameter^48^. Although we observed reduced sclerenchyma wall thickness in the mutant plant, this effect might be enough to confer structural strength to the vessel cells at least at the first development stages of the plant under hydrated (normal humidity) conditions, allowing the plants to reach an initial growth. The heterogeneities in cell wall thickness are best pictured by implosion resistance parameter distributions (Fig. 6), displaying narrower distributions for the mutant cells than for the wild type cells. The combination of a smaller average cell wall thickness with a narrower dispersion provides the conditions for cell implosion. Our data suggest that the decrease of the total lignin in the C4H mutant is accompanied by a thinning of the cell wall thickness and a narrowing of its distribution, which influences the mechanical properties corresponding to the mutant vessel collapse under normal pressures. This three-dimensional plant-imaging study enables the visualisation of different cellular hierarchical structures, such as cell walls, cytoplasm and organelles, and provides a quantitative analysis that reveals the correlation between the cell wall thickness with cell wall thickness distribution and drought resistance in plants.

In summary, we demonstrated the ability of cryo X-ray ptychography for bio-imaging by revealing the three-dimensional plant cell wall structure of *Arabidopsis thaliana* tissue on a multiscale domain. Reducing the radiation damage effect is essential for providing high-resolution images of plant tissues, which is achieved here by performing X-ray cryo imaging. With the advent of upgraded and fourth-generation synchrotron sources based on multi-bend achromat lattices, such as SIRIUS facility^49^, the coherent flux available in the hard X-ray regime is expected to increase by two orders of magnitude in the near future^50^. Combined with fast detection systems, this analysis will permit the three-dimensional imaging of larger specimens, reaching close to nanometric resolution and reducing the total data acquisition time. Finally, the tremendous potential of spectro-ptychography^51,52^ (the combination of imaging with spectroscopic capabilities) will allow studies of nanoparticle biodistribution and the elucidation of nanoparticle biokinetics and bioactivity within intact tissues in plants and animals.

## Methods

### Sample selection and tissue preparation

Seeds of the C4H *Arabidopsis thaliana* mutant were obtained from the *Arabidopsis* Biological Resource Centre, at the Ohio State University. C4H mutant and wild type of *A. thaliana* cv. Columbia plants were grown at a temperature of 22 °C with a 16 h light/8 h dark photoperiod for 45 days. The petiole fragments were selected for these studies and manually cut down to volumes of around 0.5 ×0.5 ×1 mm^3^. These fragments were fixed in 3% (v/v) glutaraldehyde diluted in cacodylate buffer (0.2 M pH 7.25) for 24 hours. The sample was post- fixed with 1% (w/v) osmium tetroxide (OsO_4_) in aqueous solution for 12 h at 4 °C. The samples were dehydrated by ascending alcohol series from 10% (v/v) to 100% (v/v) infiltrated in a hydrophilic acrylic resin (LR White^®^ “Hard Grade”; EMS). The resin polymerization took place with 100% (v/v) LR White^®^ resin for 12 h at 60 °C, in gelatine capsules.

### Scanning electron microscopy

The same tissue region studied in the X-ray measurements, from both, wild type and C4H mutant, were manually selected and cut down to smaller fragments with the aid of a razor blade. Next, the fragments were coated with gold layer using a vacuum sputter-coater to improve the sample conductivity and imaged in the JSM 5800LV (Jeol, Tokyo, Japan) microscope available at the Electron Microscopy Laboratory from Biology Institute (Unicamp, Campinas, Brazil). The microscope operated at 10 kV and images were acquired in different magnifications.

### GaFIB-SEM shaping and sample mounting into the OMNY chamber

First, we manually removed the resin excess around the sample within the resin block using a blade, thereby leaving the sample very close to the block surface. The block was attached to a scanning electron microscopy (SEM) stub using conductive carbon cement and then it was sputter-coated with carbon. Even after montage into the SEM- stub (see Supplementary Fig. S5a) the blocks needed to be trimmed with a microtome to remove empty resin and guarantee that the ROI was on the surface from which the pillars would be shaped. Therefore, the fixed blocks needed to have a firm fixture with a Silver Conductive Epoxy from CW (Circuit Works), a strong highly conductive 2-component epoxy adhesive cured at 60 °C overnight. The block surface was imaged in a Ga FIB-SEM (FEI Helios 600i) (see Supplementary Fig. S5b) from the Scientific Centre for Optical and Electron Microscopy (ScopeM-ETH Zurich) with 20 kV electrons to initially localize the region of interest (see Supplementary Fig. S5c). The ROI cylinders of 50 µm diameter by 50 µm height were generated by the Ga ion milling (see Supplementary Fig. S5d,e). The pillar was detached from the block through an undercut with the ion beam and later, with a micromanipulator, it was transferred onto a flat Au coated OMNY pin^53^, where it was attached by carbon deposition. The final pillar can be seen in Supplementary Fig. 5f. The pins containing the samples were transferred to the OMNY instrument through a modified commercial vacuum cryo transfer system (Leica EM VCT100) in liquid nitrogen^34^.

### Ptychographic X-ray computed tomography (PXCT) measurements

The experiment was done at the cSAXS beamline of the Swiss Light Source (SLS) at the Paul Scherrer Institute (Villigen PSI, Switzerland). We use the “OMNY” (“tOMography Nano crYo”) stage, an instrument that allows cryo-PXCT of biological materials^34^. The instrument comprises high-resolution 3D scanning setup, which allows nanometre precision positioning via differential interferometry and allows sample rotation over 360°. The illumination on the sample was defined by a Fresnel zone plate (FZP) of 200 µm diameter with outermost width of 60 nm, which has a focal distance of 60 mm at 6.2 keV. The FZP provided a coherent illumination with 10^8^ photons/s. The sample was placed 3 mm downstream the focus where the x-ray spot size was 10 µm. In order to reduce air scattering and absorption a helium flight tube was placed between the OMNY instrument and the Eiger 500 K detector with 75×75 µm^2^ pixel size^54^. The sample to detector distance was 7.3 m. Ptychographic scans were performed in Fermat spiral^55^ with a step size of 2.5 µm over a field of view of 60 µm x 25 µm (diameter x height) with a total number of 238 points/each angular orientation, i.e., tomographic projection. Each scanning point was exposed for 0.1 s. The total angular coverage was from 0° to 180° with angular step of 0.48°, resulting in 750 projections.

### Phase retrieval and 3D image reconstruction

For each projection, 2D diffraction patterns were recorded. An iterative phase retrieval algorithm is used to recover the 2D complex-valued transmission function of the specimen at each angular position, resulting in a reconstruction pixel size of 48.87 nm. The reconstructions were performed using the difference map algorithm^56^, followed by 100 iterations of a maximum likelihood refinement^28^. Phase projections were further processed to remove a phase off-set and a linear phase term, which are intrinsic degrees of freedom for ptychographic reconstruction, and alignment in *x* and *y* directions. The projections were also aligned with an iterative refinement using tomographic consistency^57^. The refinement alignment in *x* based on tomographic consistency was further applied. Tomographic reconstructions were performed from wrapped phase projections by a modified filtered back projection (FBP)^58^. In the FBP filtering step a Hanning low pass filter was applied with a cut-off at 50% of the Nyquist frequency. To estimate the 3D resolution, we reconstructed 2 independent tomograms, each from one half of the projections. From this reconstructions we computed the 3D Fourier shell correlation (FSC) curve and compared it with the half-bit threshold curve^35^.

### Image analysis

The images segmentation was initially performed using the Trainable Weka Segmentation (TWS) machine learning tool^36^ available in Fiji^59^ to separate the different cellular compartments. In TWS, the 3D input images were submitted to pixel classification where a set of input pixels were manually selected and attributed to a specific class, either cell wall or lumen. The selected classified pixels, i.e. the classifiers, are used to automatically classify the rest of the pixels of the complete images, based on grey value level, texture, morphology, among other features. The same classifiers trained in the wild type plant image were applied to C4H plant image.

The program output was two separate grey level images for both, the cell wall and for the lumen. Using Avizo (Thermo Scientific™), the separated images of the cellular compartments could be easily segmented by threshold based on grey value level and converted into labels. The generated label images from cell wall and lumen were refined with morphological filters. The quantitative analysis of local thickness 3D, which computes the diameter of the largest sphere that fits inside the object^37^, was applied to the cell wall and lumen labelled images, using Thickness Map module from Avizo.

To compute the implosion resistance parameter (*t*/*b*)^2^ along the vessel, the tomograms of both labels were virtually sliced at each cellular height, generating 2D images. The lumen diameter and cell thickness of each image were determined using the Euclidean distance transform algorithm (EDT)^44^ (Fig. 4). In the case of the lumen, the algorithm sets to each pixel the minimum distance of that pixel to all border pixels of the object of interest. The centre of the object is taken to be the position of the maximum of the distance transform data. Finally, the diameter is measured by acquiring the distance of the centre pixel to all the border pixels. The cell wall thickness was measured through a similar procedure. The thinning process was applied to extract the medial axis of the wall images. The thickness was obtained by measuring the distance of the medial axis pixels to the border pixels using the EDT data. The used algorithm was developed in collaboration with the LEAR group (IFSC/USP) using Python 3.4 with Skimage package.

## Supplementary information

Supplementary information.

Supplementary Video S1.

Supplementary Video S2.

Supplementary Video S3.

Supplementary Video S4.

Supplementary Video S5.

Supplementary Video S6.

## Data Availability

The data reported in this article are available from the corresponding authors upon reasonable request.
