# Supplementary material for: Correlations between lignin content and structural robustness in plants revealed by X-ray ptychography
Source: Sci Rep. 2020 Apr 7;10:6023. doi: 10.1038/s41598-020-63093-6 (PMC7138792; doi:10.1038/s41598-020-63093-6)
Supplement: Supplementary file 1 — Supplementary information. [file 41598_2020_63093_MOESM1_ESM.pdf]

Supplementary information

Correlations between lignin content and structural robustness in plants revealed by X-ray ptychography

C. C. Polo*, L. Pereira, P. Mazzafera, D. N. A. Flores-Borges, J. L. S. Mayer, M. Guizar-Sicairos, M. Holler, M. Barsi-Andreeta, H. Westfahl Jr., F. Meneau*

[1]Brazilian Synchrotron Light Laboratory (LNLS), Brazilian Center for Research in Energy and Materials (CNPEM), 13083-970, Campinas, SP, Brazil

[2]Laboratory of Plant Physiology "Coaracy M. Franco", Center R&D in Ecophysiology and Biophysics, Agronomic Institute (IAC), Campinas SP, Brazil

[3]Department of Plant Biology, Institute of Biology, P.O. Box 6109, University of Campinas (UNICAMP), 13083-970, Campinas, SP, Brazil

[4]Departament of Crop Science, College of Agriculture "Luiz de Queiroz", University of São Paulo (ESALQ-USP), CP 09, 13418-900, Piracicaba, SP, Brazil

[5]Paul Scherrer Institute, Villigen PSI, Switzerland

[6]São Carlos Institute of Physics, University of São Paulo, PO Box 369, 13560-970, São Carlos, SP, Brazil.

*Corresponding authors. E-mail: carla.polo@lnls.br and florian.meneau@lnls.br

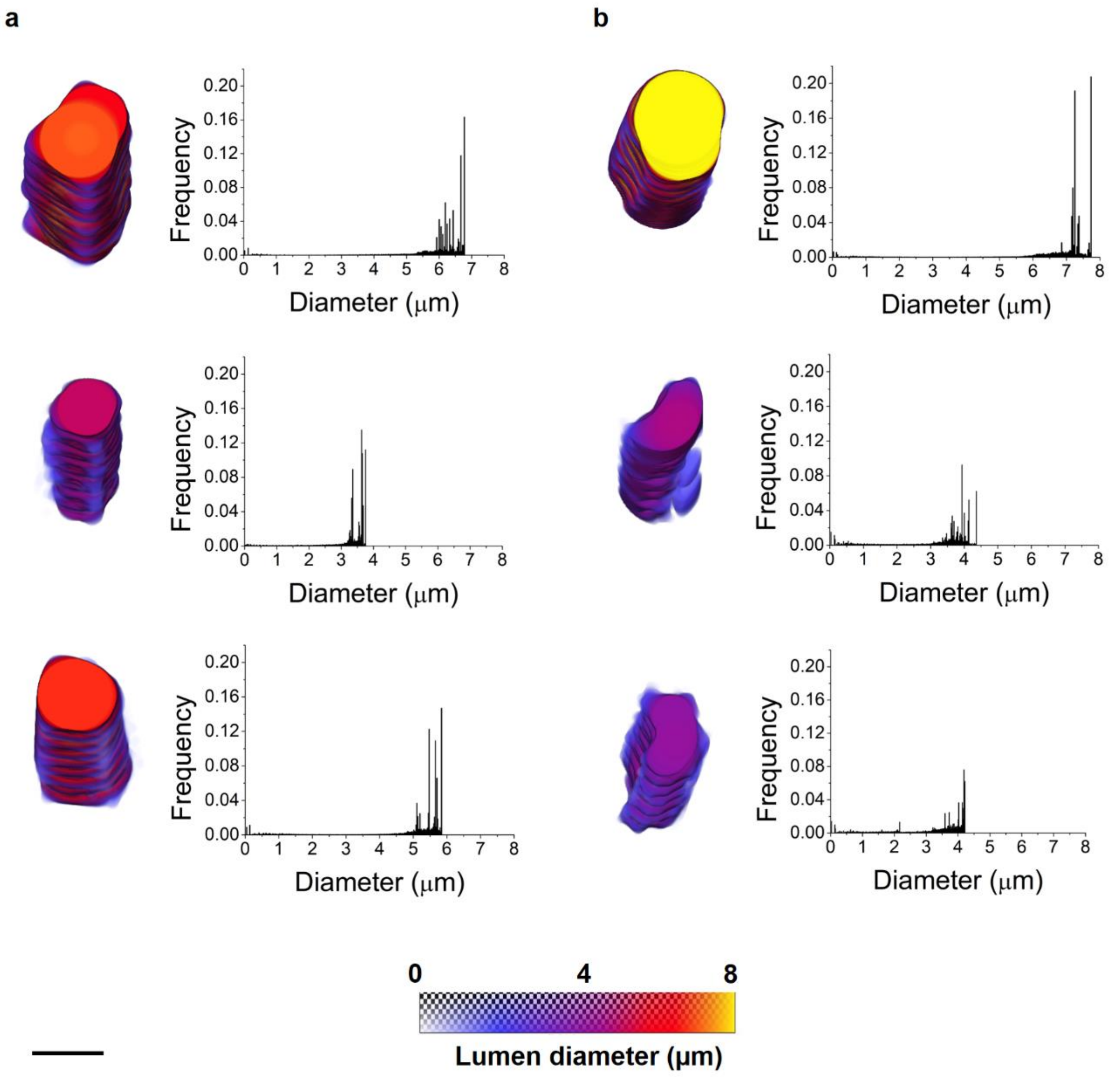

Supplementary Figure S1. Lumen diameter distribution of vessel cells. Analysis of the wild type, **a**, and C4H mutant, **b**, cells allowed to observe the distribution and evaluate the differences in diameter between the two plants. The rendering with colour map of each analysed cell (*N*=3) represents the three-dimensional maps of the diameter distribution (scale bar = 5 μm). The colour map goes from smaller thickness (violet) to larger thickness (yellow), with the values, in micrometres, represented on the top, highlighting the difference between wild type and mutant.

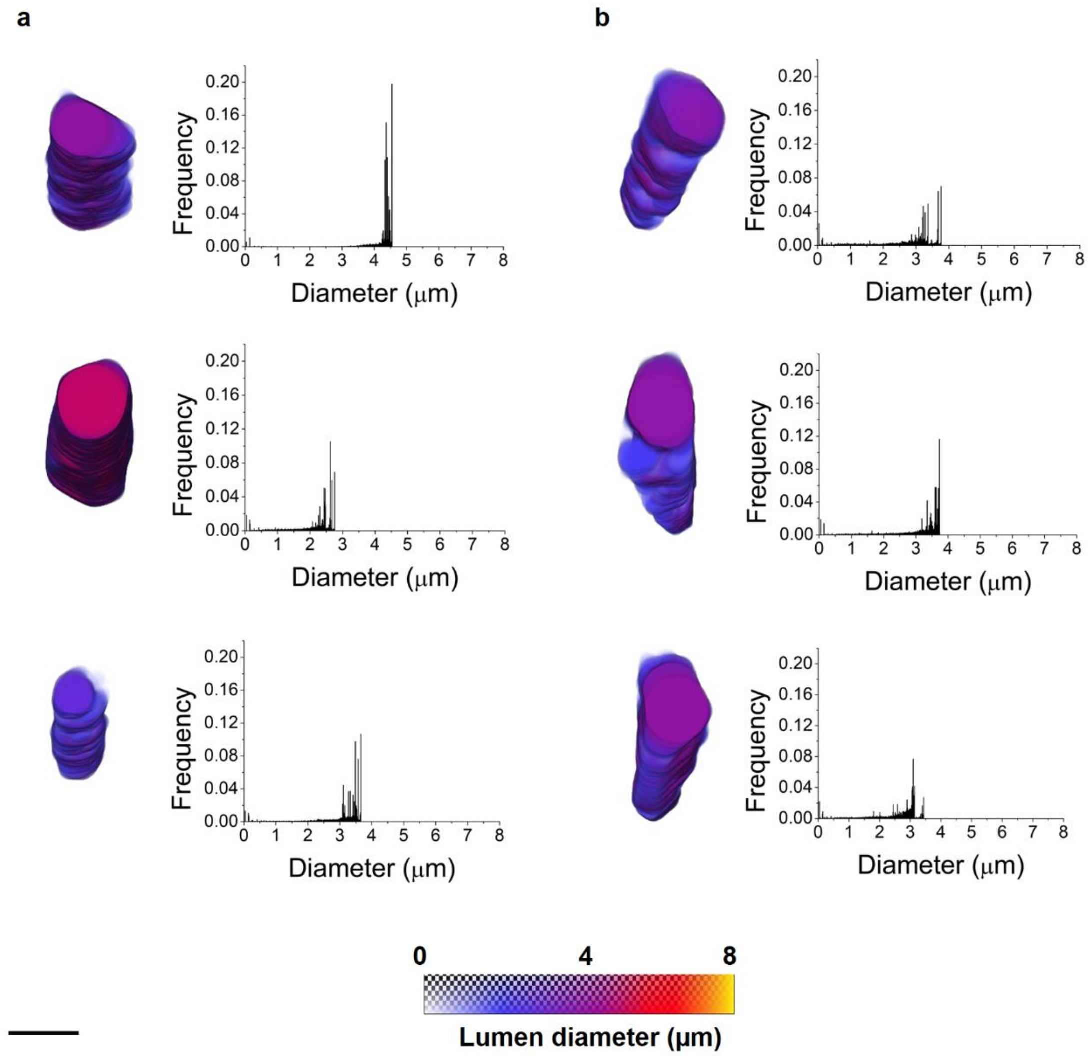

Supplementary Figure S2. Lumen diameter distribution of sclerenchyma cells. Analysis of the wild type, **a**, and C4H mutant, **b**, cells allowed to observe the distribution and evaluate the differences in diameter between the two plants. The rendering with colour map of each analysed cell (N=3) represents the three-dimensional maps of the diameter distribution (scale bar = 5 μm). The colour map goes from smaller thickness (violet) to larger thickness (yellow), with the values, in micrometres, represented on the top, highlighting the difference between wild type and mutant.

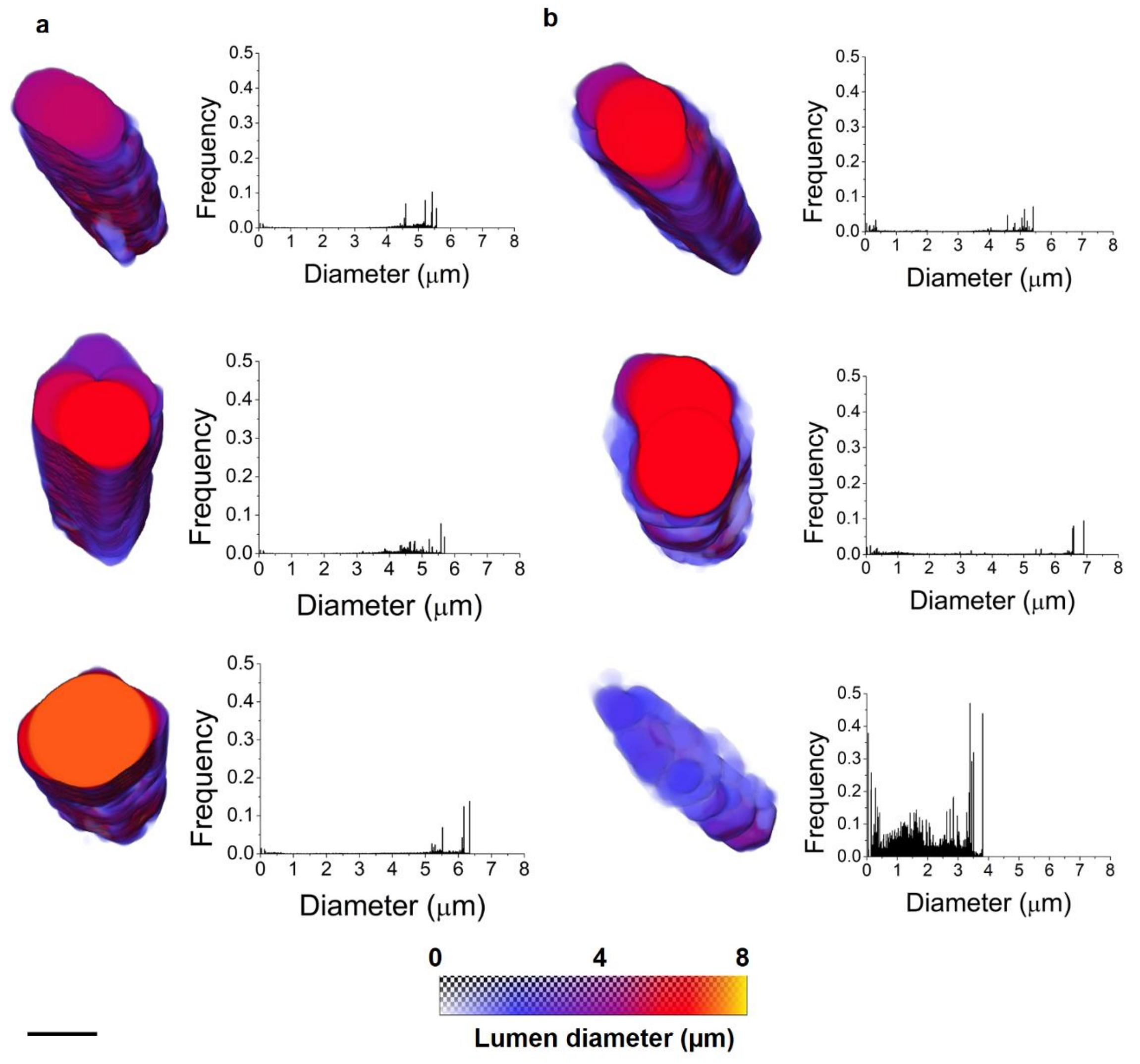

Supplementary Figure S3. Lumen diameter distribution of parenchyma cells. Analysis of the wild type, **a**, and C4H mutant, **b**, cells allowed to observe the distribution and evaluate the differences in diameter between the two plants. The rendering with colour map of each analysed cell (*N*=3) represents the three-dimensional maps of the diameter distribution (scale bar = 5 μm). The colour map goes from smaller thickness (violet) to larger thickness (yellow), with the values, in micrometres, represented on the top, highlighting the difference between wild type and mutant.

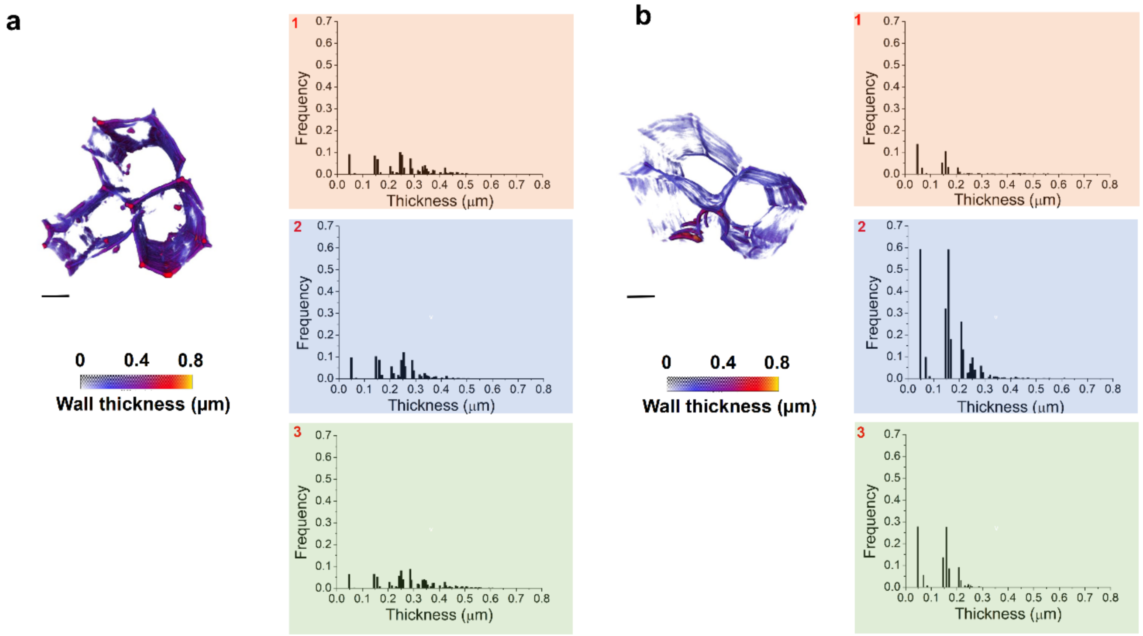

Supplementary Figure S4. Cell wall thickness distribution of parenchyma cells. Analysis of the wild type, **a**, and C4H mutant, **b**, cells allowed to observe the wall distribution and evaluate the differences in thickness between the two plants. The rendering with colour map of each cell block represents the three-dimensional maps of the thickness distribution along the cell wall volume (scale bar = 5 µm). The colour map goes from smaller thickness (violet) to larger thickness (yellow), with the values, in micrometres, represented on the top, highlighting the difference between wild type and mutant. The wall thickness analysis of each cell numbered from 1 to 3 correspond the histograms of thickness *versus* frequency.

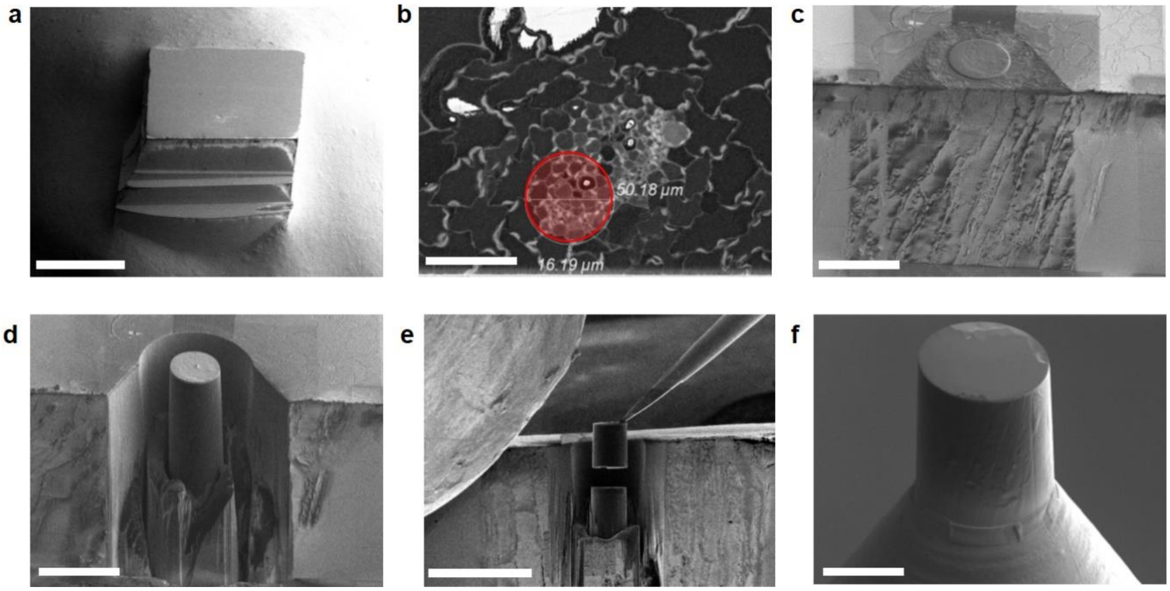

Supplementary Figure S5. Sample preparation by GaFIB-SEM. **a**, SEM surface image of the resin block containing chemically fixed sample (scale bar = 300 µm). **b**, ROI localization into the block surface, indicated by the red circle (scale bar = 50 µm). **c**, Initial pillar milling (scale bar = 75 µm). **d**, Final pillar milling (scale bar = 75 µm). **e**, Through the micromanipulator device within the equipment, the 50 µm diameter x 50 µm height pillar is detached from the block (scale bar = 150 µm). **f**, the pillar is transferred and glued to the OMNY[1] pins which will be loaded into the OMNY instrument [2](scale bar = 20 µm).

Supplementary Reference

1. Holler, M. *et al.* OMNY PIN - A versatile sample holder for tomographic measurements at room and cryogenic temperatures. *Rev. Sci. Instrum.* **88,** (2017).
2. Holler, M. *et al.* OMNY - A tOMography Nano crYo stage. *Rev. Sci. Instrum.* **89,** (2018).

Supplementary Video

Supplementary Video S1. Vessel cells rendering from wild-type plant

Supplementary Video S2. Vessel cells rendering from C4H mutant plant

Supplementary Video S3. Sclerenchyma cells rendering from wild-type plant

Supplementary Video S4. Sclerenchyma cells rendering from C4H mutant plant

Supplementary Video S5. Parenchyma cells rendering from wild-type plant

Supplementary Video S6. Parenchyma cells rendering from C4H mutant plant
